# Psychological Outcomes and Mechanisms of Mindfulness-Based Training for Generalised Anxiety Disorder: A Systematic Review and Meta-Analysis

**DOI:** 10.1007/s12144-023-04695-x

**Published:** 2023-05-11

**Authors:** Monique Williams, Cynthia Honan, Sarah Skromanis, Ben Sanderson, Allison J. Matthews

**Affiliations:** 1 School of Psychological Sciences, College of Health and Medicine, University of Tasmania, Private Bag 30, Hobart, TAS 7001 Australia; 2 School of Psychological Sciences, College of Health and Medicine, University of Tasmania, Launceston, TAS 7250 Australia

**Keywords:** Anxiety, Anxiety disorders, Attention, Mindfulness, Meditation

## Abstract

This systematic review aimed to identify 1) the effect of mindfulness training on pre-post measures of anxiety and attention among adults experiencing high levels of generalised anxiety; and 2) the impact of predictors, mediators and moderators on post-intervention changes in anxiety or attention. Trait mindfulness and distress measures were included as secondary outcomes. A systematic search was conducted in November 2021 in electronic databases using relevant search terms. Eight articles comprising four independent studies were included (*N* = 334). All studies included participants diagnosed with generalised anxiety disorder (GAD) who participated in an 8-week manualised program. The meta-analysis indicated that mindfulness training had a large effect on anxiety symptoms (*g* = -1.92, 95%*CI*[-3.44, -0.40]) when compared to inactive (i.e., care as usual, waitlist) or non-specified (i.e., condition not defined) controls. However, a significant effect was not found when compared to active controls. Effects for depression, worry and trait mindfulness did not reach statistical significance, despite small-large effect sizes favouring mindfulness compared to inactive/non-specified controls. Our narrative review found evidence that changes in aspects of trait mindfulness mediate anxiety reduction following mindfulness training. However, a small number of studies were available for inclusion in the review, with high risk of bias and low certainty of evidence present. Overall, the findings support the use of mindfulness training programs for GAD and indicate mechanisms that may differ from those involved in other cognitive therapy approaches. Further RCTs with evidence-based controls are needed to clarify techniques most beneficial for generalised anxiety to support individually tailored treatment.

**Supplementary Information:**

The online version contains supplementary material available at 10.1007/s12144-023-04695-x.

Generalised anxiety disorder (GAD) is characterised by persistent anxiety and worry about multiple areas of life and typically includes co-occurring difficulties such as tension, sleep disruption, restlessness, exhaustion, and irritability (APA, 2013; Newman et al., 2013). The course of GAD tends to be chronic (Weisberg, 2009; Wittchen, 2002), with approximately 6% of the population experiencing GAD during their lifetime (ABS, 2008; Newman et al., 2013). However, sub-clinical levels of GAD symptoms are twice as prevalent, typically persistent, and are also associated with ongoing engagement with primary health care (Haller et al., 2014). While Cognitive Behavioural Therapy (CBT) is currently the gold standard treatment for GAD (NICE, 2020), up to approximately 50% of patients with an anxiety disorder do not reach full remission (Springer et al., 2018). Further, factors such as financial cost, delivery mode, and client treatment preference present as barriers to accessing this treatment or other face-to-face therapies (Andersson & Titov, 2014; Wolitzky-Taylor et al., 2018). More recently, following COVID-19, there has been a surge in anxiety-related symptoms coupled with treatment access barriers (COVID-19 Mental Disorders Collaborators, 2021; WHO, 2020, 2022). Mindfulness-based inventions offer an alternative to CBT and involves practices that are accessible and affordable (e.g., mindfulness meditation apps; see Gál et al., 2021). Mindfulness meditation practices involve paying deliberate attention to the present moment with a non-judgemental attitude (Kabat-Zinn, 1994; Tang et al., 2015). Mindfulness-based interventions have been shown to reduce symptoms of GAD and other anxiety disorders (e.g., Ghahari et al., 2020; Haller et al., 2021; Strauss et al., 2014; VØllestad et al., 2012). However, there is a lack of clear evidence explaining underlying mechanisms of mindfulness-based interventions among those experiencing GAD or sub-clinical levels of GAD symptoms. Such knowledge is critical to ongoing treatment innovations for GAD symptoms, particularly in our changing social environment.

Several theories have been proposed to explain the aetiology of generalised anxiety. The cognitive model posits that four main components are involved: intolerance of uncertainty, positive beliefs about worry, negative problem orientation (i.e., perceived threat of problems and doubt about coping ability) and cognitive avoidance (i.e., strategies to avoid undesirable thoughts and subsequent emotions) (Dugas et al., 2007). From a biological perspective, generalised anxiety symptoms have been linked to hypoactivation of the anterior cingulate cortex (ACC) and prefrontal cortex (PFC) in response to perceived threat, coupled with amygdala hyperactivity (Brooks & Stein, 2015; Holzschneider & Mulert, 2011). According to attentional control theory, anxious individuals have increased automatic attentional processing (or orienting of attention) to threat and decreased voluntary processing (or executive control over attention) in response to threat (Eysenck et al., 2007). This is thought to produce *attentional biases to threat* relative to neutral stimuli, observable in cognitive reaction time tasks (see Bar-Haim et al., 2007; Eysenck et al., 2007). Furthermore, attention is thought to be a key mechanism involved in mindfulness, and overlap can be seen between the attentional processes that are proposed to be disrupted in anxiety and trained through mindfulness practice (Chiesa et al., 2011; Hölzel et al., 2011; Lao et al., 2016; Tang et al., 2015). Previous reviews have examined the effect of mindfulness training on attention measures in predominantly healthy or non-clinical samples, finding mixed results (Chiesa et al., 2011; Lao et al., 2016; Yakobi et al., 2021). However, no review has examined the effect of mindfulness training on attentional measures in samples experiencing high levels of generalised anxiety.

The Default Mode Network (DMN) has also been implicated in the aetiology of anxiety. The DMN comprises mid-line brain regions, including the medial prefrontal cortex (MPFC) and the posterior cingulate cortex (PCC), and is thought to be involved in mind wandering and self-referential processing (Scheibner et al., 2017). For example, mindful attention during meditation is associated with reduced DMN activity as compared to periods of self-reported mind wandering (Scheibner et al., 2017). Individuals with anxiety disorders or high levels of anxiety symptoms have shown hypoconnectivity between limbic areas with the DMN and the executive control network (involved in top-down attention and cognitive control) (Xu et al., 2019), as well as other abnormalities in the DMN and salience network (involved in the detection of relevant or salient stimuli) (Imperatori et al., 2019; Xiong et al., 2020). While previous studies have examined biological measures related to attention and anxiety processes following mindfulness-based intervention in individuals experiencing generalised anxiety symptoms, no study has systematically reviewed such findings.

Previous systematic reviews/meta-analyses have examined mediators and moderators of psychological outcomes following mindfulness interventions (e.g., Alsubaie et al., 2017; Gu et al., 2015; Johannsen et al., 2022; VØllestad et al., 2012). Trait mindfulness (i.e., general mindful ability in everyday life) has consistently been found to mediate psychological outcomes following mindfulness training (Alsubaie et al., 2017; Gu et al., 2015; Johannsen et al., 2022). However, these publications have reviewed studies comprising of non-specified samples or participants with mixed anxiety disorders, and largely involved mindfulness-based manualised programs (e.g., Mindfulness-Based Cognitive Therapy [MBCT], Mindfulness-Based Stress Reduction [MBSR]). These programs comprise other features which may exert therapeutic benefits (e.g., group setting, therapist/instructor contact, psychoeducation, cognitive behavioural therapy elements). This makes it difficult to ascertain the elements responsible for the effects of the treatment and to evaluate the effectiveness of mindfulness practice as a ‘stand-alone’ intervention. Existing systematic reviews\meta-analyses looking at the effect of ‘stand-alone’ mindfulness interventions have assessed anxiety and depression symptoms in a mixture of clinical and non-clinical samples and have not systematically examined individual characteristics that predict or moderate outcomes (Blanck et al., 2018).

The current review aimed to deepen understanding of mechanisms involved in mindfulness-based interventions for samples experiencing generalised anxiety symptoms (clinical and sub-clinical levels). Such a review is critical to the further development of accessible and effective treatment options for this population. The outcomes reviewed included anxiety symptom and attention measures (primary outcomes), as well as measures of trait mindfulness and other forms of distress (e.g., worry, depression) (treated as secondary outcomes). Additionally, we sought to review previous findings of predictors, mediators, and moderators, as well as conduct our own meta-analyses to explore the relationship between other predictor variables (e.g., personality, cognitive, biological measures) with our primary outcomes.

## Objectives

The aim of this study was to systematically review and meta-analyse the quantitative literature to answer the following questions:

1. What is the effect of mindfulness-based training interventions on pre-post measures of anxiety symptoms and attention compared to control interventions/conditions in individuals experiencing high levels of anxiety?
2. What is the impact of other variables (e.g., predictors, mediators, and moderators) on pre-post reductions in anxiety symptoms and improvements in attention following mindfulness-based training interventions in individuals experiencing high levels of anxiety?

## Methods

This systematic review was conducted in accordance with the Preferred Reporting Items for Systematic Reviews and Meta-Analyses (PRISMA) guidelines (Page et al., 2021) and Cochrane recommendations (Higgins et al., 2022). A protocol was pre-registered at PROSPERO (CRD42021222389).

### Eligibility Criteria

#### Population

Participants aged between 17 and 70 years, and assessed as having high levels of general, generalised or trait anxiety (e.g., cut-off on validated self-report measure) or a GAD diagnosis (e.g., DSM-5 assessment) were included in this review. Studies were excluded if participants were selected based on another type of anxiety (e.g., panic disorder) or psychological disorder (e.g., depression), recent or current pregnancy, or a current or previous medical, developmental, or neuropsychological condition.

#### Intervention/Comparator

Mindfulness-based training interventions needed to comprise at least two separate sessions over a specified period. Both manualised programs (e.g., MBSR, MBCT) and ‘stand-alone’ mindfulness interventions comprising isolated and repeated practice of mindfulness exercises (e.g., body scan, mindful breathing) were eligible for inclusion. Interventions were considered in light of practising at least some of the core characteristics of mindfulness specified in Kabat-Zinn’s (1994, p.4) definition: “paying attention…on purpose, in the present moment, and non-judgementally”. Only interventions including a substantial mindfulness component (i.e., > 70% mindfulness practice) were included. Interventions largely comprising other treatment approaches (e.g., relaxation, CBT, Dialectical Behaviour Therapy, Acceptance and Commitment Therapy) or non-mindfulness therapeutic elements (e.g., self-compassion, yoga, problem solving skills) were excluded. Studies were included if they included a waitlist, active, or alternative treatment control or another comparison, but were excluded if they exclusively used another mindfulness training intervention as a comparison (e.g., different or enhanced version).

#### Outcomes

Included studies needed to have reported at least one anxiety or attention outcome (i.e., primary outcomes) that was measured at both pre- and post-intervention. Anxiety-related outcomes could include self-reported symptoms (i.e., questionnaire measure), biological (e.g., functional magnetic resonance imaging [fMRI] for amygdala activation, cortisol), or physiological (e.g., skin conductance response, heart rate) measures. Attention-related outcomes could include behavioural (e.g., reaction time, accuracy), clinician-rated assessment, electroencephalography (EEG), event-related potentials (ERP), or fMRI measures of attention processes (e.g., orienting, executive control, attentional bias to threat).

#### Study Design and Characteristics

Studies were also excluded if they were not (1) a full text study article (e.g., review, conference abstract), (2) written in English or (3) published in a peer-reviewed journal. The peer-review status of eligible articles was verified using Ulrichs Web Directory (ProQuest, 2022) and Clarivate Journal Citation Reports (Clarivate, 2022). No date limits were imposed.

### Information Sources and Search Strategy

Three databases (Web of Science, Scopus, PsycINFO via Ovid) were searched, with the last search conducted on 25^th^ November 2021. We also reviewed citations from recent meta-analyses and systematic reviews related to the current topic as well as citations from studies that were included in the current review. The full search strategy was developed in consultation with an independent research Librarian at the University of Tasmania and was tested, revised and reviewed by MW. Search terms for three concepts were used: *anxiety* (“anxiety” OR “anxious”), *mindfulness* (“mindful*” OR "focused breathing" OR "breath focus" OR "raisin exercise" OR "breathing space" OR "present moment awareness" OR "body scan") and *intervention* (“treatment” OR “intervention” OR “therapy” OR “program” OR “practice” OR “induction” OR “strategy” OR “technique” OR “training” OR “psychotherapy” OR “acute” OR “brief” OR “session” OR “exercise”). The adapted search strategy for each database is provided in Supplementary Materials, Table S1. The search was conducted as part of a broader review also looking at the effect of acute mindfulness induction. However, only studies involving mindfulness-based training interventions were included in the current review.

### Selection Process

Title/abstract and full-text screening was undertaken by the reviewers using Covidence (Veritas Health Innovation, 2022), an internet-based software program. MW screened all titles/abstracts and full-text studies. Duplicates were removed and independent screening was divided between AM, SS, and BS at both title/abstract and full-text screening stages. Reviewer disagreements were discussed until consensus was reached. Where necessary, a senior author (AM) was consulted to make a final decision.

### Data Collection Process

MW extracted data for all included studies using a data extraction form created on Covidence. The form was initially piloted by MW and reviewed by a senior author (AM). AM was consulted where there were uncertainties regarding data needed for extraction and attempts were made to contact study authors for further details where information was missing or unclear. Journal titles, study authors and institutions were not blinded during extraction.

### Data Items

In addition to primary outcomes (anxiety and attention), data from self-report measures of trait mindfulness and distress was also extracted. For each study outcome, the data needed to compute standardised effect sizes for pre-post intervention was extracted for both the intervention and control/comparison groups. Where reported data compatible with an outcome domain was insufficient for pooling (i.e., less than two studies reporting same outcome measure), key findings, as defined by the study authors with respect to their hypotheses, were obtained for inclusion in the narrative review. Additionally, where regression, mediation or moderation was undertaken with anxiety or attention outcome measures, we extracted key findings, as defined by the study authors with respect to their hypotheses, for inclusion in the narrative review.

We also extracted information regarding: (1) article: author, year, journal of publication; (2) participants: sample characteristics, definition and criteria used for anxiety; (3) intervention and control groups: type, dosage, mode of delivery, adherence; (4) outcome characteristics: instruments used, type of predictors, mediators or moderators; (5) research design and features: study aims and conclusions, conflicts of interest, funding, recruitment approach, analyses undertaken.

### Study Risk of Bias Assessment

For randomised controlled trials, risk of bias was assessed using the revised Cochrane ‘Risk of bias’ tool for randomised trials (RoB 2.0) (Sterne et al., 2019). RoB 2.0 addresses five specific sources of bias that arise from: (1) the randomisation process; (2) deviations from intended interventions; (3) missing outcome data; (4) measurement of the outcome; and (5) selection of the reported result. A 'Risk of bias' (RoB) judgment was made for each domain (low; some concerns; high). For non-randomised controlled trials, The Risk of Bias in Non-randomized Studies – of Interventions (ROBINS-I) assessment tool (Sterne et al., 2016) was used. ROBINS-I addresses seven specific sources of bias that arise from: (1) confounding; (2) selection of participants; (3) classification of interventions; (4) deviations from intended intervention; (5) missing data; (6) measurement of outcomes; (7) selection of the reported result. A 'Risk of bias' (RoB) judgment was made for each domain (low; moderate; serious; critical). For each of the tools, an overall summary RoB judgement was derived for each specific outcome for each article, whereby the overall RoB was determined by the highest RoB level in any of the domains. However, where RoB was assessed as high for the ‘measurement of the outcome’ domain due to inclusion of a self-report measure and an intervention that participants were not blinded to, we chose to override the default judgement of overall high RoB. We took this approach as self-report measures are most common in clinical trials for anxiety symptoms and it is often impossible to blind a participant to a behavioural intervention. MW applied the appropriate tool to each included study, for each reported outcome. A senior author (AM) was consulted where uncertainties arose. Attempts were made to contact corresponding authors where information was missing or unclear.

### Data Synthesis Methods

#### Overall Analyses

For Objective 1 (i.e., pre-post intervention effects), standardised mean difference (SMD) effect sizes (Hedge’s *g*) between the mindfulness and control conditions were calculated with 95% confidence intervals. A negative SMD indicated greater effect of the treatment compared to control condition for anxiety, depression and worry outcomes, while a positive SMD indicated a greater effect for the trait mindfulness outcome. The effect sizes of SMDs were interpreted according to Cohen’s (1992) guidelines (0.20 = small, 0.50 = moderate, 0.80 = large). To calculate SMDs, we used pre-post change means and change standard deviations of each intervention condition. Where a study did not report pre-post change deviations for each intervention condition, and we could not obtain these from authors, these were either calculated or imputed from other available statistics, using the methods described in the Cochrane Handbook (Higgins et al., 2022).

If at least two studies reported the same pre-post outcome, pairwise meta-analyses using random-effects models with restricted maximum-likelihood were conducted. A random-effects model was chosen to calculate the average distribution of treatment effects, to account for likely variation (e.g., according to age, sex, etc.) (Higgins et al., 2022). Meta-analyses were undertaken using the *metan* package in StataSE 17 statistical software (StataCorp, 2021).

Studies with active and inactive/non-specified controls were analysed in separate meta-analyses to account for likely heterogeneity in effects due to comparison type. Relevant effects that were not eligible for inclusion in a meta-analysis (i.e., less than two studies with the same outcome measure) were reported as part of the narrative review. Both summary statistics and effect estimates were reported where sufficient data was available. Effect sizes for bivariate correlations associated with any correlational/regression analyses were interpreted according to Cohen’s (1988) guidelines (0.10 = small, 0.30 = moderate, 0.50 = large). In line with Objective 2, we sought to examine the relationship between other predictor variables (e.g., personality, cognitive, biological measures) with our primary outcomes using meta-regression. However, we encountered a paucity of data whereby the number of separate included studies containing data for the same predictor and outcome measures were too low to warrant meta-regression (i.e., less than 10; Higgins et al., 2022). Therefore, to address Objective 2, only findings from previous regression/correlational, moderation, and mediation analyses were reported in the narrative review.

#### Statistical Heterogeneity

Between-study heterogeneity was assessed using the *I*^*2*^ statistic (proportion of between-study heterogeneity) and the *Q*-statistic (whether between-study heterogeneity is greater than that expected by chance). The *T*^*2*^ statistic (including 95% CIs) was also reported as a measure of the variance of effect sizes. The magnitude of heterogeneity was categorised according to the following cut-offs: *I*^*2*^ > 30% = moderate, *I*^*2*^ > 50% = substantial, *I*^*2*^ > 75% = considerable (Higgins et al., 2003, 2022). It was not possible to perform sub-group analyses or meta-regression to explore high heterogeneity as the number of studies were too low (i.e., less than 10; Higgins et al., 2022). Where high heterogeneity was identified (> 50% or *p* < 0.05) and there were more than two studies, sensitivity analyses were performed through the removal of clear outliers, as visually inspected on forests plots.

#### Bias Assessment

To assess publication bias, we planned to visually inspect funnel plots and conduct Egger’s test. However, the number of studies were too low to perform these (i.e., less than 10 studies for a given meta-analysis; Egger et al., 1997; Higgins et al., 2022).

#### Certainty Assessment

The quality of evidence for all outcomes was evaluated by MW using the Grading of Recommendations Assessment, Development and Evaluation method using methods described in the Cochrane Handbook (Higgins et al., 2022) and by the GRADE Working Group (2022) across the following domains: risk of bias, inconsistency, indirectness, imprecision, and publication bias. The certainty of evidence was assessed as high, moderate, low, or very low. As applicable, the presence of a large effect, dose–response gradient, and plausible confounding effect were considered in upgrading the certainty of evidence.

## Results

### Study Selection

**Fig. 1:**
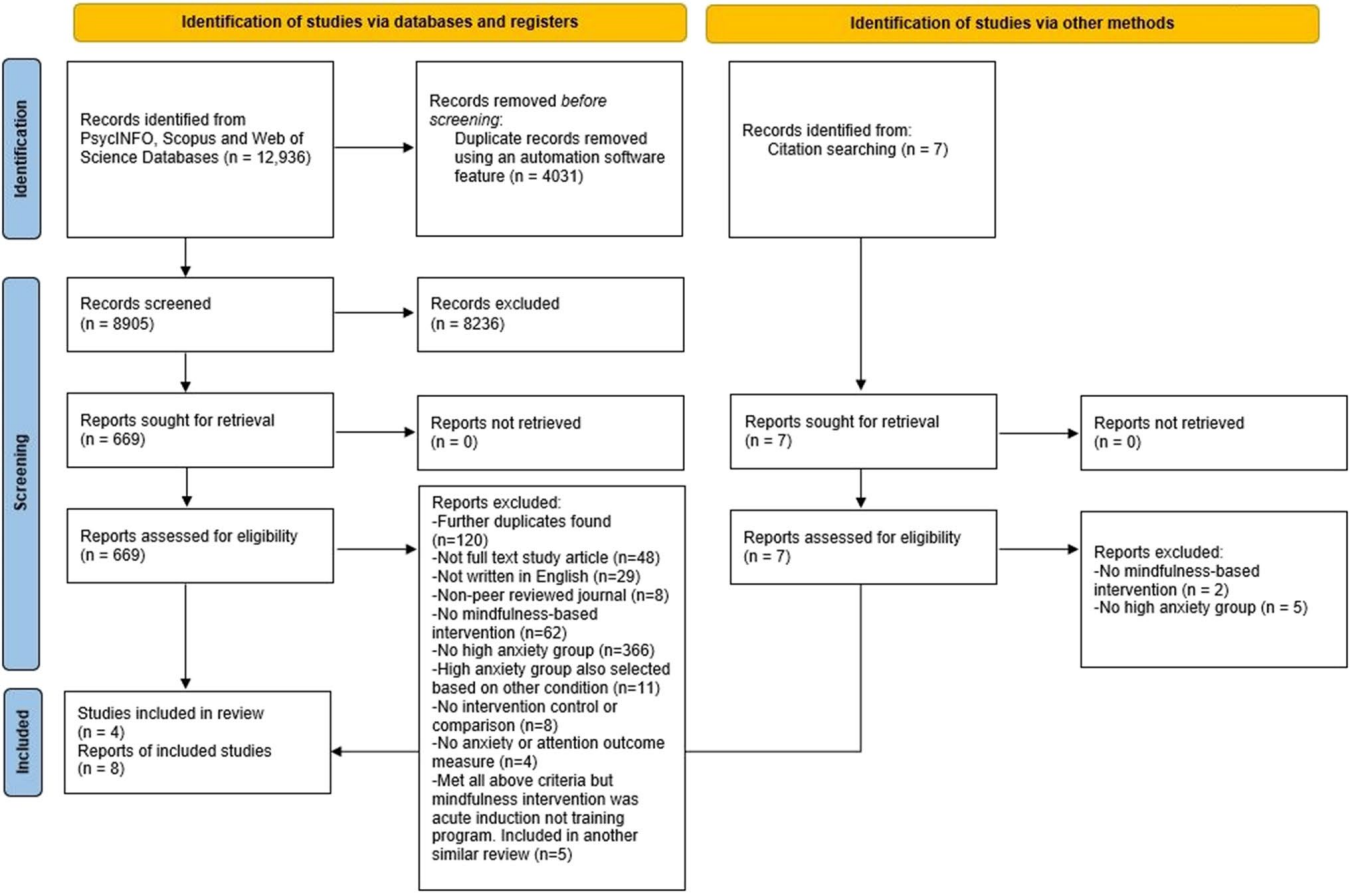
PRISMA Flow Diagram of the Record Selection Process

See Fig. 1 for a PRISMA flow chart showing the record selection process.

We found 12,936 records in databases using our search strategy. After initial automated duplicate removal in Covidence, we screened 8,905 records, from which we reviewed 669 full-text documents. While eight papers were included (see Table 1), five of these (i.e., Hoge et al., 2013, 2015, 2018, 2020; Hölzel et al., 2013) pertained to one ‘parent’ study in which samples were not independent across the five papers. Therefore, a total of four independent studies were included. Reference searches among included studies and 11 systematic reviews/meta-analyses of mindfulness interventions published in the last 10 years did not reveal additional eligible studies. Two articles (i.e., Boettcher et al., 2014; VØllestad et al., 2011) were initially included but were later excluded after data for participants with generalised anxiety could not be retrieved from the authors.

**Table 1:** Characteristics of Included Articles

Author	Origin	Sample size(*N*)	Mean age (*SD*)	Females %	Anxiety characteristics	Comorbidities	Mindfulness experience	Design	Timepoints	Anxiety outcomes	Attention outcomes	Secondary outcomes
Hoge et al., 2013	USA	89	39.0(13.9)	51%	GAD diagnosis (SCID)	12.4% depression 7.9% panic disorder 28.1% social anxiety disorder	Excluded if had more than four meditation classes (including yoga and tai chi) in past two years	RCT	Pre, post	BAI HAMA (structured interview)		
Hoge et al., 2015	USA	38	37.6(11.7)	55%	GAD diagnosis (SCID)	Not reported	As above	RCT	Pre, post	BAI		Worry: PSWQ Trait mindfulness: FFMQ Decentering: EQ (decentering subscale)
Hoge et al., 2018	USA	70	39.0(11.6)	46%	GAD diagnosis (SCID)	14.3% depression	As above	RCT	Pre, post	Cortisol ACTH IL-6 TNF-alpha		
Hoge et al., 2020	USA	42	41.9(14.5)	40%	GAD diagnosis (SCID)	Not reported	As above	Reported single arm from broader RCT	Pre, post	BAI		Trait mindfulness: FFMQ
Hölzel et al., 2013	USA	26	37.9(12.2)	54%	GAD diagnosis (SCID)	15.4% depression 3.8% panic disorder 23.1% social anxiety disorder	As above	RCT	Pre, post	BAI fMRI (amygdala)	fMRI (frontal cortex regions)	
AsmaeeMajid et al. 2012	Iran	31	32.2(2.2)	0%	GAD diagnosis (SCID) Hospital patients	Not reported	Not reported	RCT	Pre, post	BAI		Worry: PSWQ Depression: BDI
Wong et al., 2016	China	182	50.0(10.0)	79%	GAD diagnosis (SCID)	All participants reported clinically relevant depressive symptoms (> 16 on CES-D)	Excluded on current or previous regular meditation or yoga practice	RCT	Pre, post, 3-month FU, 6-month FU, 9-month FU	BAI		Worry: PSWQ Depression: CES-D Trait mindfulness: FFMQ
Zhao et al., 2019	China	32	33.6(7.7)	75%	GAD diagnosis Hospital outpatients	Not reported	Not reported	Wait-list control (Within-subjects)	Pre-waitlist, post-waitlist, post-MBCT	HAMA	fMRI (DMN: ACC, PCC, insula, MCC)	Depression: HAMD Trait mindfulness: FFMQ

*Note.* USA = United States of America; GAD = Generalized Anxiety Disorder; SCID = Structured Clinical Interview for DSM-IV; RCT = Randomised controlled trial; BAI = Beck Anxiety Inventory; HAMA = Hamilton Anxiety Rating Scale; PSWQ = Penn State Worry Questionnaire; FFMQ = Five Facet Mindfulness Questionnaire; EQ = Experiences Questionnaire; ACTH = Adrenocorticotropic hormone; IL-6 = Interleukin 6; TNF-alpha = Tumour Necrosis Factor alpha; fMRI = Functional magnetic resonance imaging; BDI = Beck Depression Inventory; CED-D = Center for Epidemiological Studies-Depression Scale; FU = Follow up; MBCT = Mindfulness Based Cognitive Therapy; DMN = Default mode network; ACC = Anterior cingulate cortex; PCC = Posterior cingulate cortex; MCC = Mid-cingulate cortex

### Study Characteristics

The eight included articles (see Table 1) were published between 2012 to 2020 and included a total of 334 independent participants with a median age of 36.31 and a median of 63% females. While samples experiencing both clinical and sub-clinical levels of generalised anxiety symptoms were eligible, all included studies involved participants who met diagnostic criteria for GAD. Details of training interventions used in each of the included studies are presented in Table 2. While both manualised and stand-alone interventions were eligible, the four independent studies all included an 8-week, group-based, manualised, mindfulness training program (two MBSR and two MBCT). Two of the four independent studies included a psychoeducation program as an active control, while three studies included a comparison group that was either inactive (care as usual/no specific intervention, within-subjects waitlist) or non-specified (details of the control condition were not reported). Five articles reported receiving funding while three reported a potential conflict of interest (details are presented in Supplementary Materials, Table S2).

**Table 2:** Characteristics of Interventions of Included Studies

Author	Mindfulness training intervention	Content of intervention	Modifications	Delivery	Frequency and dosage	Adherence	Control condition
Hoge et al., 2013, 2015, 2018, 2020; Hölzel et al., 2013	8-week MBSR	Practice of exercises to build present moment awareness with a non-judgemental attitude	Shorter times were used compared to the original program for classes (2.5 to 2 h), retreat (8 to 4 h) and homework (45 to 20 min). Metta (loving kindness) was added to the first class and homework practice	Group classes taught by MBSR instructor with > 8 years experience	8 × weekly 2-h group classes + 4-h retreat + 20 min daily homework practice	No adherence data reported for Hoge et al., 2013, 2015, 2018, 2020 Hölzel et al., 2013 (*N* = 26) Homework mins of practice MBSR: *M* = 1,116, *SD* = 499 Control: *M* = 868, *SD* = 413 Number of classes attended MBSR: *M* = 7.27, *SD* = 0.59 Control: *M* = 7.00, *SD* = 0.59 Number of participants who missed the retreat day MBSR: 26.7% Control: 9.1% No significant group differences found for any measure	8-week stress management education class active control. 8 × weekly 2-h group classes + 4-h special class + 20 min daily homework practice. Content included psychological and physical health information and gentle exercises. Delivered by an instructor with 9 years experience and a physical therapist with 22 years experience
Asmaee Majid et al., 2012	8-week MBSR	As above	Not reported	Group classes delivered by an experienced MBSR instructor	8 × weekly 2-h group classes + 30 min daily homework practice	No adherence data reported	Details of control condition not reported
Wong et al., 2016	8-week MBCT	Exercises to increase present moment awareness of feelings and thoughts and integrate mindfulness skills into daily activities	Cognitive–behavioural components related to anxiety were introduced to replace those previously relating to depression (i.e., theory and education regarding automatic thoughts, avoidance, worrying, action planning, relapse prevention)	Group classes taught by a clinical psychologist and social worker with experience in delivering MBCT	8 × weekly 2-h group classes + 45 min daily homework practice	Number of classes attended MBCT: *M* = 6.4, *SD* = 1.9 Psychoeducation group: *M* = 7.1, *SD* = 1.5 No significant group differences found in terms of completion. No baseline covariate was found to be associated with compliance (i.e., attendance of > 80% of classes)	1) 8-week Psycho-education group. 8 × weekly 2-h group classes taught by two clinical psychologists with at least two years experience. CBT-informed psychoeducation: stress, panic, insomnia, depression, thinking and behaviours 2) Care as usual. No specific intervention but allowed to access to primary care services (i.e., GP, seek referral to mental health specialist)
Zhao et al., 2019	8-week MBCT	As above	Modified psychoeducation and relapse prevention content to be relevant to GAD	Group classes taught by a psychologist with > 5 years experience in delivering mindfulness meditation	8 × weekly 2-h group classes + 30 min daily homework practice	No adherence data reported	8-week pre-intervention waitlist (within-subjects)

*Note*. MBSR = Mindfulness Based Stress Reduction; *M* = Mean; *SD* = Standard Deviation; MBCT = Mindfulness Based Cognitive Therapy; CBT = Cognitive Behavioural Therapy; GP = General practitioner

### Risk of Bias in Studies

**Fig. 2:**
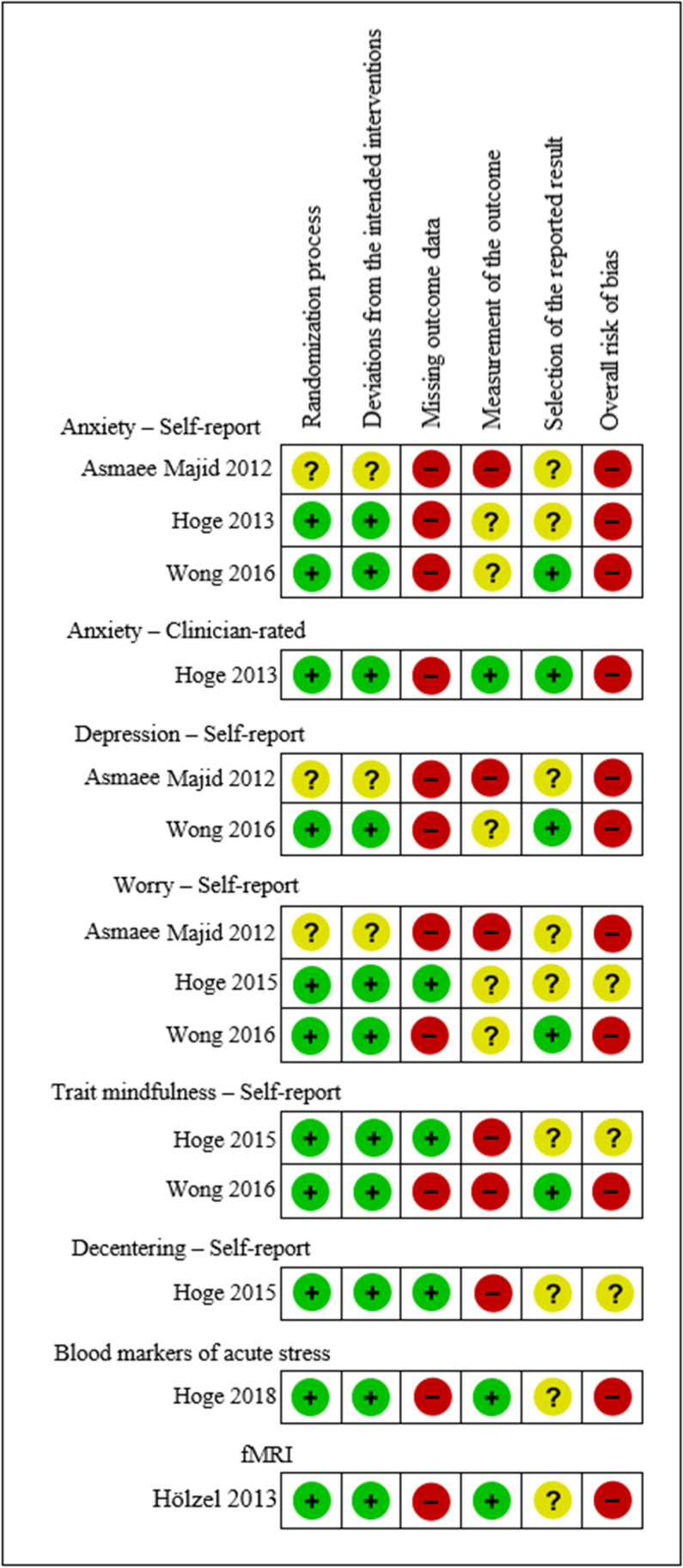
RoB 2.0 Risk of Bias Summary for Articles included in the Meta-Analysis and Narrative Review for each Outcome. *Note*. RoB assessments shown for Wong et al. (2016) are for MBCT vs psychoeducation (active control) comparisons. For MBCT vs treatment as usual comparisons for Wong et al., RoB was assessed as high for ‘measurement of the outcome’ across all outcomes, as differences in self-report bias due to knowledge of intervention are expected to be larger

The RoB 2.0 tool was applied to each outcome of interest for six (of eight) articles that reported pre-post findings for an RCT (see Fig. 2). Five of six articles were assessed as having an overall high RoB. Five of six articles were assessed as having high RoB for ‘missing outcome data’, where missingness likely depended on the true outcome value, or where there was a lack of information to rule this out. RoB was assessed as high for ‘measurement of the outcome’ for one study where it was unclear whether the control group was active and therefore controlled for self-report biases (Asmaee Majid et al., 2012). RoB was also assessed as high for ‘measurement of the outcome’ for two other articles, with outcomes deemed to be susceptible to self-report bias when mindfulness training is compared to an active control not containing mindfulness (i.e., trait mindfulness, decentering [i.e., ability to observe thoughts and feelings with distance] outcomes). One article did not clearly report a random allocation sequence or enough information to determine whether an appropriate analysis was used. We were not able to obtain an a priori analysis plan for one of the articles, and the plan for four other articles lacked sufficient information, leading to ‘some concerns’. The ROBINS-I was completed for one non-randomised study (Zhao et al., 2019; see Table 3). Results were comparable with RCTs in terms of measurement of outcome and selection of reported results. Confounding was judged as expected, but adequately measured and controlled for. The final article included in this review (Hoge et al., 2020) was part of a broader ‘parent’ RCT where intervention effects for included measures were examined in other included articles. As only results for the treatment group and the mediation analyses were reported in this article, a RoB assessment was not applied.

**Table 3:** ROBINS-I Risk of Bias Assessment for One Non-Randomised Study included in the Meta-Analysis (Anxiety, Depression, Trait Mindfulness) and Narrative Review (fMRI)

	Outcome	Domain 1	Domain 2	Domain 3	Domain 4	Domain 5	Domain 6	Domain 7	Overall
Zhao 2019	Anxiety (self-report)	Moderate	Low	Low	Low	Low	Serious	No information	Moderate
	Depression (self-report)	Moderate	Low	Low	Low	Low	Serious	No information	Moderate
	Trait Mindfulness (self-report)	Moderate	Low	Low	Low	Low	Serious	No information	Moderate
	fMRI	Moderate	Low	Low	Low	Low	Low	No information	Moderate

*Note*. Domain 1 = bias due to confounding; Domain 2 = bias in selection of participants into the study; Domain 3 = bias in classification of interventions; Domain 4 = bias due to deviations from intended interventions; Domain 5 = bias due to missing data; Domain 6 = bias in measurement of outcomes; Domain 7 = bias in selection of the reported result; fMRI = Functional magnetic resonance imaging

### Results of Syntheses

#### Pre-Post Effects for Self-Reported Anxiety Symptoms

**Fig. 3:**
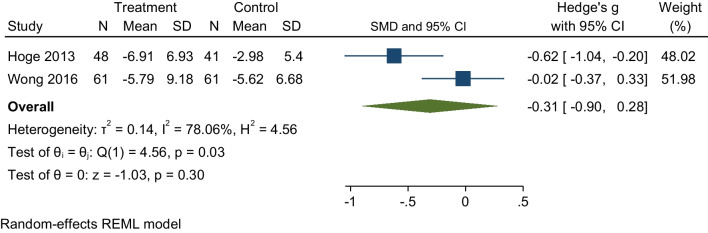
Forest Plot Showing the Effect of Manualised Mindfulness Programs on Anxiety Symptoms as Compared to Active Control Groups

Results from two RCTs including active controls (psychoeducation related) were pooled (see Fig. 3). The mean pre-post change in anxiety was not statistically significant following the mindfulness programs compared to the psychoeducation programs (2 trials; *n* = 211; *SMD* = -0.31, 95%*CI*[-0.90, 0.28], 95%*PI*[0.00, 0.00], *p* = 0.302). However, there was evidence of considerable heterogeneity ($x$^*2*^ = 4.56, *p* = 0.033, *τ*^2^ = 0.14, *I*^*2*^ = 78%).

**Fig. 4:**
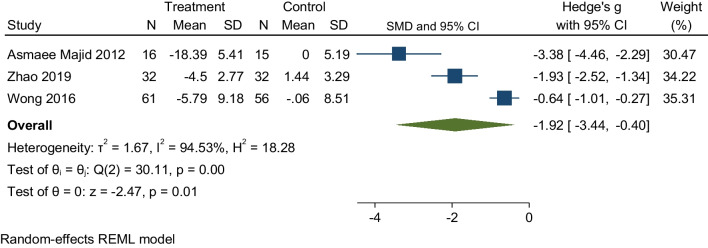
Forest Plot Showing the Effect of Manualised Mindfulness Programs on Anxiety Symptoms as Compared to Inactive/Non-Specified Control Groups

Results from three studies including inactive/non-specified controls were pooled (see Fig. 4). Greater reductions in anxiety were observed following mindfulness programs as compared to inactive/non-specified control groups (3 trials; *n* = 212; *SMD* = -1.92, 95%*CI*[-3.44, -0.40], 95%*PI*[-21.06, 17.23], *p* = 0.014). However, there was evidence of considerable heterogeneity ($x$^*2*^ = 30.11, *p* < 0.001, *τ*^2^ = 1.67, *I*^*2*^ = 95%).

One RCT used a clinician-rated measure of anxiety (Structured Interview Guide for the HAMA; not included in meta-analysis) (Hoge et al., 2013) and found a statistically significant reduction in anxiety symptoms following both MBSR (*p* < 0.001) and a stress management education active control (*p* < 0.001). There was not a significant group*time interaction, and the size of the difference in effect between groups was small (*g* = -0.28, 95%*CI*[-0.13, 0.70]). Summary statistics and effect estimates are presented in Supplementary Materials, Table S3. One RCT (Wong et al., 2016) reported reductions in self-reported anxiety at 3-month follow-up for both MBCT and psychoeducation relative to care as usual (*p* < 0.05). Reductions were maintained at both 6-month and 9-month follow-up (*p* < 0.05), and there were no differences between interventions at any timepoint (*p* > 0.05).

#### Pre-Post Effects for Self-Reported Depression Symptoms

**Fig. 5:**
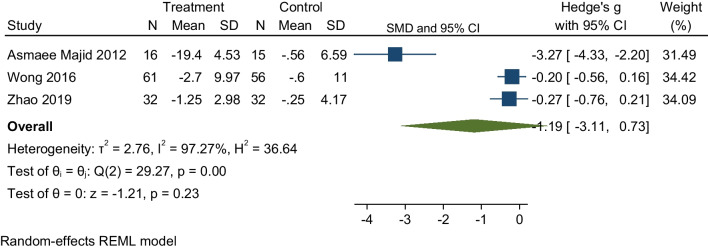
Forest Plot Showing the Effect of Manualised Mindfulness Programs on Depression Symptoms as Compared to Inactive/Non-Specified Control Groups

Results from three studies with inactive/non-specified controls were pooled (see Fig. 5). The mean pre-post change in depression was not statistically significant following the mindfulness programs compared to the non-active/non-specified control groups (3 trials; *n* = 212; *SMD* = -1.19, 95%*CI*[-3.11, 0.73], 95%*PI*[-25.71, 23.33], *p* = 0.225). However, there was evidence of considerable heterogeneity ($x$^*2*^ = 29.27, *p* < 0.001, *τ*^2^ = 2.76, *I*^*2*^ = 97%). There was one clear outlier (i.e., Asmaee Majid et al., 2012) that had lower RoB across domains and did not report details of the control group. Removal of this outlier (forest plot provided in Supplementary Materials, Figure S1) did not lead to a statistically significant effect of mindfulness programs compared to control groups (2 trials; *n* = 181; *SMD* = -0.23, 95%*CI*[-0.52, 0.07], 95%*PI*[0.00, 0.00], *p* = 0.128), but there was no longer evidence of heterogeneity ($x$^*2*^ = 0.06, *p* = 0.812, *τ*^2^ < 0.01, *I*^*2*^ = 0%).

Wong et al. (2016) included a second control group that was active (psychoeducation). Reductions in depression did not significantly differ following MBCT compared to psychoeducation (*p* > 0.05), despite a small-moderate effect size favouring psychoeducation (*g* = 0.45, 95%*CI*[0.10, 0.81]). Summary statistics and effect estimates are presented in Supplementary Materials, Table S3. Wong et al. also assessed self-reported depression at follow-up. For both MBCT and psychoeducation conditions, reductions in depression were found at both 6-month and 9-month follow-up (*p* < 0.05), with no differences found between MBCT and psychoeducation or care as usual at any time point (*p* > 0.05).

#### Pre-Post Effects for Self-Reported Worry

Results from two RCTs with active controls were pooled. The mean pre-post change in worry was not statistically significantly different following the mindfulness programs compared to the active control groups (2 trials; *n* = 160; *SMD* = 0.07, 95%*CI*[-0.26, 0.40], 95%*PI*[0.00, 0.00], *p* = 0.665). No evidence of heterogeneity was observed ($x$^*2*^ = 1.08, *p* = 0.299, *τ*^2^ = 0.01, *I*^*2*^ = 7%). Results from two RCTs with inactive/non-specified controls were pooled. The mean pre-post change in worry was not statistically significant following the mindfulness programs compared to inactive/non-specified control groups (2 trials; *n* = 148; *SMD* = -2.27, 95%*CI*[-6.32, 1.79], 95%*PI*[0.00, 0.00], *p* = 0.273). However, there was evidence of considerable heterogeneity ($x$^*2*^ = 36.69, *p* < 0.001, *τ*^2^ = 8.33, *I*^*2*^ = 97%). The forest plots for the two meta-analyses are provided in Supplementary Materials, Figures S2 and S3. One RCT assessed self-reported worry at follow-up (Wong et al., 2016). For both MBCT and psychoeducation conditions, reductions in worry were maintained at both 6-month and 9-month follow-up (*p* < 0.05). No differences were found between MBCT and psychoeducation or care as usual at any time point (*p* > 0.05).

#### Pre-Post Effects for Self-Reported Trait Mindfulness

Results from two RCTs with active controls were pooled. The mean pre-post change in trait mindfulness was not statistically significant following the mindfulness programs compared to the active control groups (2 trials; *n* = 160; *SMD* = 0.16, 95%*CI*[-0.56, 0.87], 95%*PI*[0.00, 0.00], *p* = 0.666). However, there was evidence of considerable heterogeneity ($x$^*2*^ = 3.95, *p* = 0.047, *τ*^2^ = 0.20, *I*^*2*^ = 75%). Results from two studies with inactive controls were pooled. The mean pre-post change in trait mindfulness approached statistical significance following the mindfulness programs compared to the inactive control groups (2 trials; *n* = 181; *SMD* = 0.85, 95%*CI*[-0.04, 1.74], 95%*PI*[0.00, 0.00], *p* = 0.062). However, there was evidence of considerable heterogeneity ($x$^*2*^ = 7.56, *p* = 0.006, *τ*^2^ = 0.36, *I*^*2*^ = 87%). The forest plots for the two meta-analyses are provided in Supplementary Materials, Figures S4 and S5. One RCT assessed self-reported trait mindfulness at follow-up (Wong et al., 2016). For both MBCT and psychoeducation conditions, increases in trait mindfulness were maintained at 3-month, 6-month and 9-month follow-up (*p* < 0.05). No differences were found between MBCT and psychoeducation at any time point (*p* > 0.05). However, greater increases in trait mindfulness were found following MBCT relative to care as usual at 3-month follow-up (*p* < 0.05).

One RCT included the measure decentering. Hoge et al. (2015) found a significantly greater increase in decentering following MBSR as compared to a stress management education program (*p* < 0.001), with large effect (*g* = 1.31, 95%*CI*[0.62, 2.00]). Summary statistics and effect estimates are presented in Supplementary Materials, Table S3.

#### Pre-Post Effects for fMRI Measures

##### Amygdala

Hölzel et al. (2013) found a statistically significant pre-post reduction in right amygdala activation in response to neutral faces following both MBSR and a stress management education program (*p* < 0.001). However, significant changes in amygdala activation were not found following either intervention in response to happy or angry faces, and there were no significant differences between the interventions (*p* < 0.050). Hölzel et al. (2013) found a significant increase in functional connectivity between an area in the right amygdala with regions of the frontal cortex (left rostral ACC, left and right rostral middle frontal cortex, and right superior frontal cortex) following MBSR but not the stress management education program, with the connectivity changing from a negative to positive relationship at post-intervention (*p* < 0.050). Means and standard deviations for findings are not available.

##### Frontal Cortex

Hölzel et al. (2013) found a greater increase in ventrolateral PFC activity (in the right pars opercularis) in response to neutral faces following MBSR compared to stress a management education program (*p* = 0.017). Hölzel et al. (2013) also found a greater increase in right rostral middle frontal cortex (*p* = 0.020) and right pars opercularis (*p* = 0.014) activity in response to angry faces following MBSR compared to a stress management education program. Means and standard deviations for findings are not available.

##### Default Mode Network

Zhao et al. (2019) found a significant increase in PCC connectivity with the ACC and bilateral insula following MBCT (*p* < 0.050) but not following an initial waitlist control period (*p* > 0.050). Zhao et al. also found regions that overlapped in terms of reduced activity and increased DMN functional connectivity (mid-cingulate cortex [MCC], bilateral insula). Means and standard deviations for findings are not available.

#### Pre-Post Effects for Blood Markers of Acute Stress

One study included Hypothalamic–Pituitary–Adrenal Axis (HPA-axis) hormonal (cortisol and adrenocorticotropic hormone [ACTH]) and immunological (tumour necrosis factor-alpha [TNF-alpha] and interleukin-6 [IL-6]) markers of acute stress (Hoge et al., 2018). Measures were taken during a laboratory-based Trier Social Stress Test (TSST), which involves participants delivering a speech and performing arithmetic. Following MBSR relative to a stress management education program, greater reductions were found in ACTH (*p* = 0.007), TNF-alpha (*p* = 0.033) and IL-6 (*p* = 0.036), but not cortisol (*p* = 0.38). Summary statistics are presented in Supplementary Materials, Table S3.

#### Correlates of Anxiety Reduction

Hoge et al. (2015) found pre-post change in self-reported GAD symptoms was strongly and negatively associated with pre-post change in self-reported trait mindfulness (*r* = -0.54, *p* < 0.001) and decentering (*r* = -0.53, *p* < 0.001). Hölzel et al. (2013) found a significant negative relationship between pre–post activation change in the left pars triangularis of the ventral lateral PFC and change in self-reported anxiety symptoms (Spearman’s *ρ* =  − 0.62, *p* < 0.001), such that increases in activation of this area were related to decreases in anxiety symptoms. Furthermore, negative significant relationships were found between pre-post change in self-reported anxiety symptoms and pre-post change in functional connectivity between the right amygdala and the left (*ρ* =  − 0.65, *p* < 0.001) and right (*ρ* =  − 0.49, *p* = 0.018) rostral middle frontal cortex, and the right superior frontal cortex (*ρ* =  − 0.42, *p* = 0.044), such that increases in functional connectivity were related to decreases in anxiety symptoms. Further, Zhao et al. (2019) that found a positive relationship between the strength of DMN functional connectivity in the right MCC and self-reported anxiety symptoms decreased significantly from pre (*r* = 0.50, *p* < 0.010) to post-MBCT (*r* = 0.01, *p* > 0.050) (*z* = 2.04, *p* < 0.050). Effect estimates for correlational findings are presented in Supplementary Materials, Table S4.

#### Correlates of Gains in Attention

Zhao et al. (2019) found the relationship between the strength of DMN functional connectivity in the right MCC and the ‘describing’ facet of self-reported trait mindfulness changed from negative at pre-MBCT (*r* = -0.34, *p* > 0.050) to positive at post-MBCT (*r* = 0.24, *p* > 0.050) (*z* = -2.28, *p* < 0.050). Zhao et al. also found the negative relationship between the strength of DMN functional connectivity in the right MCC and the ‘non-reactivity’ facet of self-reported trait mindfulness decreased from pre-MBCT (*r* = -0.76, *p* < 0.010) to post-MBCT (*r* = -0.27, *p* > 0.050) (*z* = -2.73, *p* < 0.050). Effect estimates for correlational findings are presented in Supplementary Materials, Table S4.

#### Mediators of Anxiety Reduction

Hoge et al. (2015) found that change in both self-reported trait mindfulness (Effect estimate = 2.69, 95% CI[0.01, 8.97]) and decentering (Effect estimate = 5.84, 95% CI[2.04, 13.44]) significantly mediated the relationship between MBSR (compared to a stress management education program) and reductions in self-reported anxiety symptoms. When mediators were examined simultaneously, decentering (Effect estimate = 3.66, 95% CI[0.68, 8.38]), but not trait mindfulness (Effect estimate = 1.89, 95% CI[-0.06, 7.17]), remained statistically significant. Hoge et al. (2020) measured interpretation bias using a homophone task. The percentage of homophones (two or more words with the same pronunciation but different spelling and meaning) spelled in a threatening way (e.g., die vs dye) was taken as a measure of interpretation bias. While reductions in interpretation bias were related to reductions in self-reported anxiety symptoms following MBSR (coefficient = 0.16, *p* = 0.050), reductions in interpretation bias were not found to mediate a relationship between increases in trait mindfulness and reductions in anxiety symptoms following MBSR (Effect estimate = 0.03, 95% CI[-0.03, 0.09]).

### Certainty Assessment

Certainty of the evidence was assessed for each outcome using the GRADE rating tool (see Table 4.). Explanations for each rating judgement are provided in footnotes. Across outcomes, certainty of evidence was predominantly rated as ‘Very Low’. Evidence was largely downgraded for Risk of Bias, Inconsistency (heterogeneity in effects) and Indirectness (lack of ‘stand-alone’ interventions and samples defined by sub-clinical symptoms). While publication bias was not examined statistically due to the small number of included studies, it was not strongly suspected as we found both negative and positive trial publications and employed a comprehensive search strategy.

**Table 4.** Summary of Findings Table using the GRADE Rating System

Certainty assessment									Summary of findings			
Outcome	Number of studies	Study design	Risk of bias	Inconsistency	Indirectness	Imprecision	Publication bias	Overall certainty of evidence	Mindfulness intervention *n*	Control *n*	Relative effect (95% CI)	Absolute effect (95% CI)
Mindfulness training program vs. active control												
Self-report anxiety	2	RCT	Serious^a^	Very serious^b^	Serious^c^	Serious^d^	Not suspected^e^	⨁◯◯◯ VERY LOW	109	102	-	SMD^f^ **0.31 lower** (0.90 lower to 0.28 higher)
Clinician-rated anxiety	1	RCT	Serious^g^	N/A^h^	Serious^c^	Not serious^i^	N/A^j^	⨁⨁◯◯LOW	48	41	-	*G*^k^ **0.28 lower** (0.13 lower to 0.70 higher)
Self-report depression	1	RCT	Serious^l^	N/A^h^	Serious^c^	Not serious^i^	N/A^j^	⨁⨁◯◯LOW	61	61	-	*G*^k^ **0.45 higher** (0.10 higher to 0.81 higher)
Self-report worry	2	RCT	Serious^m^	Not serious	Serious^c^	Serious^d^	Not suspected^e^	⨁◯◯◯VERY LOW	80	80	-	SMD^f^ **0.07 higher** (0.26 lower to 0.40 higher)
Self-report trait mindfulness	2	RCT	Serious^n^	Very serious^b^	Serious^c^	Serious^d^	Not suspected^e^	⨁◯◯◯ VERY LOW	80	80	-	SMD^f^ **0.16 higher** (0.56 lower to 0.87 higher)
Self-report decentering	1	RCT	Not serious^o^	N/A^h^	Serious^c^	Not serious^i^	N/A^j^	⨁⨁⨁◯ MODERATE	19	19	-	*g*^k^ **1.31 higher** (0.62 higher to 2.00 higher)
Blood markers of acute stress	1	RCT	Serious^p^	N/A^h^	Serious^c^	Not serious^q^	N/A^j^	⨁⨁◯◯ LOW	42	28	-	Not estimable. Significant mindfulness training gains found for three measures
Amygdala fMRI	1	RCT	Serious^p^	N/A^h^	Serious^c^	Serious^r^	N/A^j^	⨁◯◯◯ VERY LOW	15	11	-	Not estimable. Significant mindfulness training gains found for amygdala connectivity with frontal regions
Frontal cortex fMRI	1	RCT	Serious^p^	N/A^h^	Serious^c^	Serious^r^	N/A^j^	⨁◯◯◯ VERY LOW	15	11	-	Not estimable. Significant mindfulness training gains found for activation in frontal regions
Mindfulness training program vs. inactive or non-specified control												
Self-report anxiety	3	RCT	Very serious^s^	Very serious^b^	Serious^c^	Not serious^i^	Not suspected^e^	⨁◯◯◯ VERY LOW	109	103	-	SMD^f^ **1.92 lower** (3.44 lower to 0.40 lower)
Self-report depression	3	RCT	Very serious^s^	Very serious^t^	Serious^c^	Serious^d^	Not suspected^e^	⨁◯◯◯ VERY LOW	109	103	-	SMD^f^ **1.19 lower** (3.11 lower to 0.73 higher)
Self-report worry	2	RCT	Very serious^s^	Very serious^b^	Serious^c^	Serious^d^	Not suspected^e^	⨁◯◯◯ VERY LOW	77	71	-	SMD^f^ **2.27 lower** (6.32 lower to 1.79 higher)
Self-report trait mindfulness	2	RCT	Serious^u^	Very serious^b^	Serious^c^	Not serious^i^	Not suspected^e^	⨁◯◯◯ VERY LOW	93	88	-	SMD^f^ **0.85 higher** (0.04 lower to 1.74 higher)
Default Mode Network fMRI	1	Non-randomised within-subjects waitlist control	Not serious^v^	N/A^h^	Serious^c^	Not serious^w^	N/A^j^	⨁⨁⨁◯ MODERATE	32	32	-	Not estimable. Significant mindfulness training gains found for connectivity within DMN regions

^a^Evidence was downgraded by 1 level because the overall risk of bias was rated as high in both studies, mainly due to missing outcome data, and there were some concerns in both studies

^b^Evidence was downgraded by 2 levels as *p* < .050 and *I*^*2*^ > 75%, indicating considerable heterogeneity

^c^Evidence was downgraded by 1 level as there was not the availability of samples defined by sub-clinical symptoms of anxiety (e.g., generalised anxiety symptoms, trait anxiety) or trials that used a non-manualised or ‘stand-alone’ mindfulness intervention

^d^Downgraded one level as pooled sample size meets power estimate but CI found to be wide (i.e., upper or lower limit cross SMD of 0.5 in both directions)

^e^Egger’s test could not be conducted but publication bias was not strongly suspected as both negative and positive trial publications were found for outcomes, and a comprehensive search for studies was employed

^f^SMD is the pooled estimate derived from the meta-analysis

^g^Evidence was downgraded by 1 level because the overall risk of bias was rated as high due to missing outcome data

^h^Only one study is available for this outcome and therefore inconsistencies cannot be considered

^i^Pooled sample size meets power estimate and CI not found to be wide (i.e., upper or lower limit do not cross SMD of 0.5 in either direction)

^j^Cannot be assessed due to availability of only 1 study

^k^*g* is derived from calculations using data available in the study article

^l^Evidence was downgraded by 1 level, mainly due to missing outcome data

^m^Evidence was downgraded by 1 level because the overall risk of bias was rated as high in one study, mainly due to missing outcome data, and there were some concerns in both studies

^n^Evidence was downgraded by 1 level because the overall risk of bias was rated as high in one study, mainly due to missing outcome data, and there were some concerns in both studies

^o^Evidence was not downgraded as risk of bias due to the lack of a pre-analysis plan was deemed unlikely to lower confidence in the estimate of effect for the outcome of interest

^p^Evidence was downgraded by 1 level, mainly due to missing outcome data

^q^Sample size meets power estimate. Numerical value for CI not available but graph in journal article shows CI is not wide (do not cross mean difference of 0.5 in either direction)

^r^CI not available but sample size does not meet power estimate

^s^Evidence was downgraded by 2 levels because the overall risk of bias was rated as high in two studies, mainly due to missing outcome data, and there were some concerns in all studies. One study had a high risk of bias or some concerns in all domains

^t^Evidence was downgraded by 2 levels as *p* < .050 and *I*^*2*^ > 75%, indicating considerable heterogeneity. When an outlier study that did not specify the control that was used was removed from the meta-analysis, heterogeneity was no longer observed

^u^Evidence was downgraded by 1 level, mainly due to missing outcome data

^v^Evidence was not downgraded as risk of bias due to confounding or the lack of a pre-analysis plan was deemed unlikely to lower confidence in the estimate of effect for the outcome of interest

^w^CI not available but sample size meets power estimate

## Discussion

Our primary aim was to review the effect of mindfulness training interventions on anxiety symptoms and attention (primary outcomes of interest), and where available, measures of trait mindfulness and distress (secondary outcomes), in samples experiencing generalised anxiety symptoms (clinical and sub-clinical). We found eight articles that met our eligibility criteria, and these comprised four independent studies. All studies included participants experiencing GAD and an 8-week manualised mindfulness program. Thus, our present findings are specific to this population and type of intervention. Pooling of three studies revealed a statistically significant reduction in self-reported anxiety symptoms following MBSR/MBCT compared to inactive/non-specified controls, with very large effect. While an overall effect was observed (i.e., no CIs crossing zero), a high level of heterogeneity was found, with a smaller effect size noted in a study with greater methodological rigour. Reductions in anxiety following MBSR/MBCT did not significantly differ when compared to active controls. Previous reviews have found moderate-large effects of mindfulness training interventions on anxiety symptoms but have tended to use mixed anxiety disorder samples or not report differences due to control type (e.g., Ghahari et al., 2020; Strauss et al., 2014; VØllestad et al., 2012). However, Haller et al. (2021) found large effects of mindfulness-based training programs on anxiety when compared to treatment as usual and moderate effects when compared to psychoeducation in samples with mixed anxiety disorders. Thus, the current findings extend on previous literature and suggest that while mindfulness programs may be effective in reducing anxiety symptoms among those experiencing GAD, they may not result in greater symptom reduction than psychoeducation programs that do not include mindfulness practices.

Our narrative review also found evidence of reductions in other indices of arousal and generalised anxiety symptoms following mindfulness training. We found evidence of reductions in hormonal (ACTH) and immunological (TNF-alpha, IL-6) markers of acute stress and increased connectivity between the amygdala and frontal cortex regions (ACC, middle frontal cortex, superior frontal cortex) following MBSR compared to active control (Hoge et al., 2018; Hölzel et al., 2013). Amygdala hyperactivity and hypoactivity in the ACC and PFC have been implicated in the development and maintenance of anxiety disorders (Brooks & Stein, 2015; Holzschneider & Mulert, 2011) and likely underly physiological reactivity and neuroendocrine changes related to both sustained and acute stress responses through the HPA-axis (Hoge et al., 2018; Patriquin & Mathew, 2017). In line with this, a recent review found functional changes have mainly been observed within amygdala-prefrontal circuits following treatment (predominantly CBT) in anxiety disorders (Baumel et al., 2022). Thus, our findings suggest that 8-week mindfulness training programs similarly target underlying brain-circuitry mechanisms involved in GAD.

We did not find any relevant studies in the literature that included cognitive or behavioural measures of attentional processes. We did, however, identify fMRI findings of functional changes in brain regions that have been implicated in attention. Increased activity in frontal cortex regions (i.e., ventrolateral PFC, middle frontal cortex) following MBSR (Hölzel et al., 2013) and increased connectivity between regions of the DMN (PCC connectivity with the ACC and insula) following MBCT (Zhao et al., 2019) were found compared to active and waitlist controls, respectively. The ACC, insula and areas of the PFC are thought to be involved in executive control attentional functions (Petersen & Posner, 2012), while the insula has also been implicated in regulatory processes such as interoceptive awareness (i.e., perception of bodily sensations) and responding to saliency (Menon & Uddin, 2010). Furthermore, the DMN has been linked to self-referential mind wandering (Scheibner et al., 2017). While previous work has shown reduced connectivity between DMN, limbic, and executive control network areas, and abnormalities in the salience network, in individuals with anxiety symptoms or disorders (Xiong et al., 2020; Xu et al., 2019), mindfulness training has been associated with improved functional connectivity within and between regions of these networks (Mooneyham et al., 2016). The current findings indicate that mindfulness training works to improve the neurocircuitry and functional activity in attention-related brain regions that may be impacted in those experiencing GAD. Further work using behavioural tasks and electrophysiological measures would help to clarify whether detriments to specific attentional processes observed in anxious individuals (e.g., executive control, attentional bias to threat) (see Bar-Haim et al., 2007; Pacheco-Unguetti et al., 2011), and proposed to be involved in GAD (Hirsch & Mathews, 2012), can be alleviated following mindfulness training.

We did not find statistically significant reductions in self-reported depression or worry following MBSR/MBCT, albeit small to large effect sizes for reductions favouring mindfulness when compared to inactive/non-specified controls. This contrasts with previous reviews showing reductions in depression in mixed anxiety disorders or non-specified samples when compared to inactive controls or no controls (moderate-large effects), as well as when compared to other active controls (small effect) (e.g., Blanck et al., 2018; Haller et al., 2021; VØllestad et al., 2012). However, Haller et al. found less reductions in depression in mixed anxiety disorders when compared to CBT. Moreover, while limited reviews have assessed the effect of mindfulness training on worry in general, several RCTs have shown similar reductions in worry following both mindfulness and active control (relaxation, working memory training) training interventions in samples experiencing high levels of worry, but not clinical levels of generalised anxiety per se (Course-Choi et al., 2017; Delgado et al., 2010). Worry and rumination are characteristic of generalised anxiety and depressive symptoms, respectively, and both involve repetitive negative thinking, leading to increases in negative affect (McLaughlin et al., 2007). Furthermore, positive beliefs about worry and tendencies to avoid unpleasant cognitions are thought to be key maintenance factors involved in GAD, both acting to provide immediate anxiety relief (Dugas et al., 2007; Newman et al., 2013). It has been suggested that mindfulness is limited as a treatment strategy for generalised anxiety as it does not challenge unhelpful beliefs (Wells, 2002). Rather, mindfulness practice encourages being open to the present moment, regardless of the associated discomfort (Baer, 2003). Our current findings suggest that mindfulness training offers limited or unreliable reduction in symptoms associated with repetitive negative thinking in GAD when compared with other interventions or no intervention.

Pooling revealed a trend towards increased trait mindfulness following MBSR/MBCT when compared to inactive, but not active, controls, with large effect. Further, our narrative review found evidence of gains in ‘decentering’ following MBSR compared to active control, with very large effect (Hoge et al., 2015). Previous work has shown acute effects of mindfulness induction on measures of state mindfulness when compared to non-therapeutic control exercises (e.g., listening to a story, thought wandering) in individuals with high levels of trait anxiety (McEvoy et al., 2017; Xu et al., 2017). It has been proposed that ongoing practice of mindfulness meditation works to improve self-regulation through the processes of attentional control, emotional regulation, and self-awareness (see Hölzel et al., 2011; Tang et al., 2015). Our findings may extend on previous work using samples experiencing milder anxiety difficulties and provide some support for this theory in individuals with GAD. However, the current findings suggest that trait mindfulness abilities may not reliably improve in individuals experiencing GAD due to mindfulness training specifically.

The second aim of the current review was to identify the impact of other variables (e.g., predictors, mediators, and moderators) on reductions in anxiety and gains in attention following mindfulness training. While limited existing findings were identified in the literature, one study found pre-post gains in decentering mediated the relationship between completion of MBSR (compared to active control) and reduced symptoms of anxiety (Hoge et al., 2015). Furthermore, another study found gains in aspects of trait mindfulness (i.e., describing, non-reactivity) were related to increases in functional connectivity in the DMN (i.e., in MCC; related to cognitive control) following MBCT compared to waitlist (Zhao et al., 2019). These findings are consistent with previous reviews that have identified trait mindfulness as a mediator of anxiety reduction following mindfulness training interventions in mixed samples (Alsubaie et al., 2017; Gu et al., 2015; Johannsen et al., 2022). The current narrative review also found that interpretation bias was not a significant mediator of the relationship between increased trait mindfulness and reductions in anxiety symptoms following MBSR (Hoge et al., 2020). Moreover, changes in negative cognitions appear to be particularly relevant mechanisms of anxiety reduction following CBT (e.g., Gallagher et al., 2020; Gómez Penedo et al., 2021; Kladnistki et al., 2022). The current findings may support the possibility that, in GAD, mindfulness exerts its effects through practice of adopting a mindful, non-judgemental, and distanced relationship with one’s thoughts and feelings, rather than challenging maladaptive thinking, as in other Cognitive Therapy approaches (Baer, 2003; Kabat-Zinn, 2003; Wells, 2002).

We note several limitations with the current body of evidence. Firstly, few independent eligible studies were identified and high risk of bias and heterogeneity were noted across studies. This contributed to overall Low-Very Low certainty of evidence ratings for the majority of outcomes. Furthermore, two of four independent studies did not report information relating to current or past meditation experience or intervention adherence, meaning these potential threats to internal validity cannot be confidently ruled out. We also note that one review member extracted data and assessed risk of bias and certainty of evidence. While a senior review author checked plausibility of decisions where there was uncertainty, we have introduced some risk of error. Nonetheless, we are confident that this limitation would not change our overall conclusions. Another limitation is that some missing data could not be obtained from authors and needed to be imputed (i.e., change standard deviations). Imputed data may therefore lack precision, leading to over/underestimation of effects. We also adopted stringent criteria regarding the degree of mindfulness practice required in training interventions (i.e., > 70%) and excluded those largely comprising other treatment approaches (e.g., self-compassion, CBT). While this enabled us to assess the effect of mindfulness-specific interventions, few studies met these criteria in addition to having a high anxiety group. In relation to objective 2 (predictors, mediators, moderators of mindfulness training outcomes in anxious individuals), the requirement for studies to have both a pre-post outcome and control group means we have likely restricted other, less rigorous, but potentially relevant studies. It is also important to note that the grey literature was not included in this review. While the requirement for articles to be peer-reviewed is a strength of our methodology, we have potentially excluded work of sufficient quality. Future research could build on our work by conducting broader reviews with more lenient criteria.

Despite limitations, the findings of the current review are of value to both healthcare providers and consumers. The findings suggest that 8-week manualised mindfulness programs may be just as effective as psychoeducation programs in reducing self-reported anxiety in GAD. While only one RCT reported follow-up measures, gains appear to be maintained for 9-months following each of these interventions. However, mindfulness-based programs appear to provide additional benefits by changing aspects of underlying central nervous system and endocrine system functioning associated with GAD (Brooks & Stein, 2015; Holzschneider & Mulert, 2011; Patriquin & Mathew, 2017). Further RCTs with follow-up assessment and objective measures are needed to evaluate potential neuroplasticity and longer-term symptom relief in GAD. In future work, inclusion of ‘stand-alone’ interventions and evidence-based treatment controls (i.e., CBT) would help to delineate the distinct therapeutic mechanisms of mindfulness-based programs. CBT-based cognitive techniques may be integral in targeting key maladaptive cognitive biases involved in GAD and worry symptoms (Dugas et al., 2007; Gómez Penedo et al., 2021; Kladnistki et al., 2022). However, specific mindfulness techniques may hold promise as treatment adjuncts. According to the contrast avoidance model of GAD, individuals over-engage in worry as a strategy to sustain distress and avoid distinct shifts in emotions that may be experienced as overwhelming (Newman et al., 2013). Potentially, mindfulness techniques focused on observing and accepting emotions may be important for therapeutic change in generalised anxiety, even if working from a predominantly CBT framework. While RCTs concerning anxiety treatment have historically been treatment framework- or program-specific, further technique- and mechanism-specific research may be most valuable to fully comprehend how second and third wave therapeutic approaches can be adequately integrated and delivered to both those experiencing clinical and sub-clinical levels of GAD symptoms. This is important as research and clinical practice looks towards more individually tailored and accessible programs to treat anxiety symptoms (Gega et al., 2022).

Our findings suggest that 8-week mindfulness training programs reduce self-reported anxiety in GAD with large effect when compared to inactive/non-specified controls, and lead to changes in underlying brain neurocircuitry (e.g., amygdala-frontal connectivity) and endocrine functioning when compared to psychoeducation programs. While we did not find any literature including behavioural or electrophysiological measures of specific attentional processes following mindfulness training, we found evidence that, in GAD, manualised mindfulness programs improve the functioning of brain regions implicated in attention. Further, the current literature suggests that gains in aspects of trait mindfulness may be important for anxiety symptom reduction following mindfulness training in GAD. Further high quality RCTs including objective measures are needed, as well as trials including ‘stand-alone’ mindfulness interventions and techniques compared to other evidence-based treatment. Such work is critical to our knowledge of mechanistic pathways to improvement and our subsequent understanding of how accessible mindfulness training can be effectively tailored to those experiencing symptoms of GAD.

## Supplementary Information

Below is the link to the electronic supplementary material.

Supplementary file1 (DOCX 64 KB)

## Data Availability

Materials, including extracted data from included studies, data used for analyses, analytic code, and other materials used in the review will be provided to researchers upon reasonable request to the corresponding author.

## Declarations

### Registration and Protocol

This systematic review/meta-analysis has been registered in the international prospective register of systematic reviews (PROSPERO) under the registration number: CRD42021222389.
