# Supplementary material for: Psychological Outcomes and Mechanisms of Mindfulness-Based Training for Generalised Anxiety Disorder: A Systematic Review and Meta-Analysis
Source: Curr Psychol. 2023 May 11:1–23. Online ahead of print. doi: 10.1007/s12144-023-04695-x (PMC10173921; doi:10.1007/s12144-023-04695-x)
Supplement: Supplementary file 1 — Supplementary file1 (DOCX 64 KB) [file 12144_2023_4695_MOESM1_ESM.docx]

##

## Supplementary Material

**Psychological Outcomes and Mechanisms of Mindfulness-Based Training for Generalised Anxiety Disorder: A Systematic Review and Meta-Analysis**

**Table S1***Free Text and Controlled Vocabulary Search Strategies for each Database*

| Database | Search strategy |
| --- | --- |
| PsycINFO | 1. anxiety.mp.  2. anxious.mp.  3. anxiety/ or anxiety sensitivity/ or anxiety disorders/ or generalized anxiety disorder/  4. 1 or 2 or 3  5. mindful*.mp.  6. focused breathing.mp.  7. breath focus.mp.  8. raisin exercise.mp.  9. breathing space.mp.  10. present moment awareness.mp.  11. body scan  12. mindfulness/  13. mindfulness-based interventions/  14. 5 or 6 or 7 or 8 or 9 or 10 or 11 or 12 or 13  15. treatment/ or psychotherapy/  16. intervention/  17. therapy.mp.  18. program.mp.  19. practice.mp.  20. induction.mp.  21. treatment.mp.  22. intervention.mp. 23. strategy.mp. 24. technique.mp. 25. training.mp. 26. psychotherapy.mp. 27. acute.mp. 28. brief.mp. 29. session.mp.  30. exercise  31. 15 or 16 or 17 or 18 or 19 or 20 or 21 or 22 or 23 or 24 or 25 or 26 or 27 or 28 or 29 or 30 32. 4 and 14 and 31 |
|  |  |
|  |  |
| Web of Science | TOPIC: (anxiety) OR TOPIC: (anxious) AND TOPIC: (mindful*) OR TOPIC: ("focused breathing") OR TOPIC: ("breath focus") OR TOPIC: ("raisin exercise") OR TOPIC: ("breathing space") OR TOPIC: ("present moment awareness") OR TOPIC: ("body scan") AND TOPIC: (treatment) OR TOPIC: (intervention) OR TOPIC: (therapy) OR TOPIC: (program) OR TOPIC: (practice) OR TOPIC: (induction) OR TOPIC: (strategy) OR TOPIC: (technique) OR TOPIC: (training) OR TOPIC: (psychotherapy) OR TOPIC: (acute) OR TOPIC: (brief) OR TOPIC: (session) OR TOPIC: (exercise) Indexes=SCI-EXPANDED, SSCI, A&HCI, CPCI-S, CPCI-SSH, ESCI, CCR-EXPANDED, IC Timespan=All years |
|  |  |
|  |  |
| Scopus | ( ( TITLE-ABS-KEY ( anxiety ) OR TITLE-ABS-KEY ( anxious ) AND ( ( TITLE-ABS-KEY ( mindful*) OR TITLE-ABS-KEY ( {focused breathing} ) OR TITLE-ABS-KEY ( {breath focus} ) OR TITLE-ABS-KEY ( {raisin exercise} ) OR TITLE-ABS-KEY ( {breathing space} ) OR TITLE-ABS-KEY ( {present moment awareness} ) OR TITLE-ABS-KEY ( {body scan} )) ) AND ( ( TITLE-ABS-KEY ( treatment ) OR TITLE-ABS-KEY ( intervention ) OR TITLE-ABS-KEY ( therapy ) OR TITLE-ABS-KEY ( program ) OR TITLE-ABS-KEY ( practice ) OR TITLE-ABS-KEY ( induction ) OR TITLE-ABS-KEY ( strategy ) OR TITLE-ABS-KEY ( technique ) OR TITLE-ABS-KEY ( training ) OR TITLE-ABS-KEY ( psychotherapy ) OR TITLE-ABS-KEY ( acute ) OR TITLE-ABS-KEY ( brief ) OR TITLE-ABS-KEY (session) OR TITLE-ABS-KEY (exercise) |
|  |  |
|  |  |

**Table S2**

*Conflicts of Interest and Funding Reported for Included Articles*

| Author | Conflict of interest | Funding |
| --- | --- | --- |
| Hoge et al. 2013 | Author 11 declared being an advisory board member, consultant, having equity in, holding royalties for and receiving research grants from several sources. | Grant from National Centre on Complementary and Alternative Medicine.  Additional support from The Highland Street Foundation. |
| Hoge et al. 2015 | Author 7 declared potential consulting relationships and receiving research grants from several sources. | As above. |
| Hoge et al. 2018 | Several authors declared receiving grants.  Author 7 declared potential consultancy, equity and royalty relationships. | As above. |
| Hoge et al. 2020 | None declared. | Grant from National Centre on Complementary and Alternative Medicine. |
| Hölzel et al. 2013 | None declared. | Grants from Marie Curie International Outgoing Fellowship within the 7th European Community Framework Programme, Varela research by the Mind and Life Institute, and the National Institutes of Health.  Research was carried out at the Athinoula A. Martinos Center for Biomedical Imaging at the Massachusetts General Hospital, using resources provided by the Center for Functional Neuroimaging Technologies.  Use of instrumentation supported by the NCRR Shared Instrumentation Grant Program. |
| Majid et al. 2012 | None declared. | None declared. |
| Wong et al. 2016 | None declared. | Grant from Health and Health Services Research Fund of the Food and Health Bureau of the HKSAR government. |
| Zhao et al. 2019 | None declared. | Grant from National Natural Science Foundation of China. |

**Table S3**

*Summary Statistics and Effect Estimates for Pre-Post Effects referred to in the Narrative Review*

|  |  |  | Treatment  (mindfulness program) | | | Control | | |  |
| --- | --- | --- | --- | --- | --- | --- | --- | --- | --- |
| Author | Comparator | Outcome instrument | *M^a^* | *SD^a^* | *N* | *M^a^* | *SD^a^* | *N* | *g*[95%*CI*s] |
| Anxiety symptoms |  |  |  |  |  |  |  |  |  |
| Hoge et al. 2013 | Stress management education program | Clinician-rated | -7.81 | 6.90 | 48 | -5.85 | 6.87 | 41 | -0.28[-0.13, 0.70] |
| Depression symptoms |  |  |  |  |  |  |  |  |  |
| Wong et al. 2016 | Psychoeducation program | Self-report | -2.70 | 9.97 | 61 | -7.92 | 12.70 | 61 | 0.45[0.10, 0.81] |
| Decentering |  |  |  |  |  |  |  |  |  |
| Hoge et al. 2015 | Stress management education program | Self-report | 8.00 | 4.80 | 19 | 2.60 | 3.10 | 19 | 1.31[0.62, 2.00] |
| Blood markers of acute stress | |  |  |  |  |  |  |  |  |
| Hoge et al. 2018 | Stress management education program | Cortisol | -152 | 256 | N/A^b^ | -89 | 328 | N/A^b^ | N/A^b^ |
| Hoge et al. 2018  Hoge et al. 2018 | Stress management education program  Stress management education program | ACTH  TNF-alpha | -290  -64 | 541  284 | N/A^b^  N/A^b^ | 200  73 | 792  188 | N/A^b^  N/A^b^ | N/A^b^  N/A^b^ |
| Hoge et al. 2018 | Stress management education program | IL-6 | -37 | 156 | N/A^b^ | 33 | 62 | N/A^b^ | N/A^b^ |

*Note*. ACTH=adrenocorticotropic hormone; TNF-alpha=tumour necrosis factor-alpha; IL-6=interleukin-6; N/A=not available
^a^ Means and standard deviations are for pre- to post-intervention change scores. Change standard deviations were calculated from *t*-statistics for Hoge et al. (2013) and were imputed for Wong et al. (2016).
^b^The exact sample sizes for each intervention group for each blood marker analysis differed, but are not reported (see Hoge et al., 2018) and thus subsequent effect sizes could not be calculated.

**Figure S1**
*Forest Plot Showing the Effect of Manualised Mindfulness Programs on Depression Symptoms as Compared to Inactive/Non-Specified Control Groups* *following removal of an Outlier*

****Figure S2**
*Forest Plot Showing the Effect of Manualised Mindfulness Programs on Worry Symptoms as Compared to Active Control Groups*

****Figure S3**
*Forest Plot Showing the Effect of Manualised Mindfulness Programs on Worry Symptoms as Compared to Inactive/Non-Specified Control Groups*

**Figure S4**
*Forest Plot Showing the Effect of Manualised Mindfulness Programs on Trait Mindfulness as Compared to Active Control Groups*

**Figure S5**
*Forest Plot Showing the Effect of Manualised Mindfulness Programs on Trait Mindfulness as Compared to Inactive Control Groups*

**Table S4***Effect Estimates for Correlational Findings*

| Author | Variable 1 | Variable 2 | Pearson’s *r* | *p*-value |
| --- | --- | --- | --- | --- |
| Hoge et al. 2015 | FFMQ Pre-post change | BAI  Pre-post change | -.54 | <.001 |
| Hoge et al. 2015 | Decentering  Pre-post change | BAI  Pre-post change | -.53 | <.001 |
| Zhao et al. 2019 | FFMQ – Describing subscale. Pre-MBCT | Strength of functional connectivity in the DMN of the right MCC. Pre-MBCT | -.34 | >.050 |
| Zhao et al. 2019 | FFMQ – Describing subscale. Post-MBCT | Strength of functional connectivity in the DMN of the right MCC. Post-MBCT | .24 | >.050 |
| Zhao et al. 2019 | FFMQ – Non-reactivity subscale. Pre-MBCT | Strength of functional connectivity in the DMN of the right MCC. Post-MBCT | -.76 | <.010 |
| Zhao et al. 2019 | FFMQ – Non-reactivity subscale. Post-MBCT | Strength of functional connectivity in the DMN of the right MCC. Post-MBCT | -.27 | >.050 |
| Zhao et al. 2019 | HAMA – Psychic anxiety subscale.  Pre-MBCT | Strength of functional connectivity in the DMN of the right MCC. Pre-MBCT | .50 | <.010 |
| Zhao et al. 2019 | HAMA – Psychic anxiety subscale.  Post-MBCT | Strength of functional connectivity in the DMN of the right MCC. Post-MBCT | .01 | >.050 |
|  |  |  | Spearman’s *ρ* |  |
| Hölzel et al. 2013 | Left pars triangularis fMRI activation. Pre-post change | BAI  Pre-post change | -.62 | <.001 |
| Hölzel et al. 2013 | Functional connectivity of the right amygdala and the left rostral middle frontal cortex. Pre-post change | BAI  Pre-post change | -.65 | <.001 |
| Hölzel et al. 2013 | Functional connectivity of the right amygdala and the right rostral middle frontal cortex. Pre-post change | BAI  Pre-post change | -.49 | .018 |
| Hölzel et al. 2013 | Functional connectivity of the right amygdala and the right superior frontal cortex. Pre-post change | BAI  Pre-post change | -.42 | .044 |

*Note*. FFMQ=Five Factor Mindfulness Questionnaire; MBCT=Mindfulness-Based Cognitive Therapy; DMN=Default Mode Network; MCC=Mid-Cingulate Cortex; HAMA=Hamilton Anxiety Inventory; fMRI=functional magnetic resonance imaging; BAI=Beck Anxiety Inventory
